# Two single amino acid substitutions in the intervening region of Newcastle disease virus HN protein attenuate viral replication and pathogenicity

**DOI:** 10.1038/srep13038

**Published:** 2015-08-12

**Authors:** Bin Liu, Yanhong Ji, Zhongqing Lin, Yuguang Fu, Rihab Muhammad Dafallah, Qiyun Zhu

**Affiliations:** 1 State Key Laboratory of Veterinary Etiological Biology, Lanzhou Veterinary Research Institute, Chinese Academy of Agricultural Sciences, Lanzhou, 730046, PR China

## Abstract

Among the proteins encoded by Newcastle disease virus (NDV), the attachment protein (HN) is an important determinant of virulence and pathogenicity. HN has been molecularly characterized at the protein level; however, the relationship between the molecular character of HN and the animal pathotype it causes has not been well explored. Here, we revisited the intervening region (IR) of the HN stalk and extended the known biological functions of HN. Three distinct substitutions (A89Q, P93A, and L94A) in the IR of genotype VII NDV (G7 strain) HN protein were analyzed. The A89Q and L94A mutations weakened the fusion promotion activity of HN to 44% and 41% of that of wild type, respectively, whereas P93A decreased the neuraminidase activity to 21% of the parental level. At the virus level, P93A and L94A-bearing viruses displayed impaired receptor recognition ability, neuraminidase activity, and fusion-promoting activity, all of which led to virus attenuation. In addition, the L94A-mutated virus showed a dramatic decline in replication and was attenuated in cells and in chickens. Our data demonstrate that the HN biological activities and functions modulated by these specific amino acids in the IR are associated with NDV replication and pathogenicity.

Newcastle disease virus (NDV) is a member of the Paramyxoviridae family, which are enveloped, non-segmented, negative-stranded RNA viruses^1^. The NDV genome is about 15 Kb and encodes six structural proteins: (in 3′ to 5′ order) nucleoprotein (NP), phosphoprotein (P), matrix (M), fusion (F), hemagglutinin-neuraminidase (HN), and large protein (L). NDV infection is initiated by receptor recognition and binding to the host cell surface, which is followed by fusion mediated by the HN and F glycoproteins. Two spikes on the NDV surface make major contributions to virulence, contagiousness, host range, and tissue tropism^2^,^3^,^4^.

The HN protein is a type II homotetrameric glycoprotein that is present on the surface of virions and infected cells. The ectodomain of HN has a globular head perched on top of a membrane-anchored stalk domain^5^,^6^. The globular head is responsible for attachment to sialic acid-containing receptor(s), whereas the neuraminidase (NA) activity of the HN protein removes sialic acid from progeny virus particles, preventing self-aggregation and unmasking the antigenic sites^7^,^8^. The stalk then interacts with the F protein to promote membrane fusion^9^,^10^,^11^.

The fusion process is transient and complex. For NDV and some other paramyxoviruses, such as parainfluenza virus 5 (PIV5), measles virus (MV), and Nipah virus (NiV), this process requires the concerted action of attachment proteins (HN/H/G) and the F protein in a virus-specific manner. The homologous HN and F proteins must be co-expressed for syncytium formation, and chimera studies have identified roles for specific domains by positioning them on the HN/H/G stalk and F head^12^. Sequence analysis of the HN stalk has identified a partially conserved segment at amino acids 74 to 110 that harbors two conserved heptad repeats (HR1 and HR2) and a seven amino acid intervening region (IR) between the HRs from residues 89 to 95^11^,^13^.

Individual amino acid substitutions in the HRs can alter the function of the HN protein^11^,^14^,^15^. The IR has two highly conserved residues among paramyxoviruses: P93 and L94. Previous studies have used site-directed mutagenesis to explore the influence of these sites^11^,^16^; however, in the context of the live Newcastle virus, the data are lacking and controversial. Currently, the A89Q-harboring Beaudette C strain (BC strain) is the only IR-mutated virus that has been successfully recovered with a modest effect on virus function; L90A-, P93A-, and L94A-HN containing viruses could not be rescued^16^. Therefore, in the present study, we chose a genotype VII NDV strain (G7), which is epidemic in China, to investigate the biological characteristics of NDV HN.

## Results

### Sequence comparison of the HN stalk and point mutations in the IR of G7

**Figure 1:**
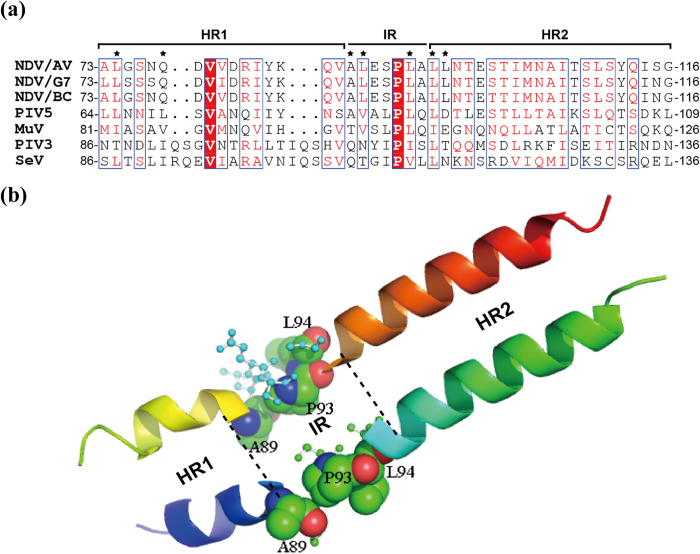
Helical structure of the HN stalk region. (**a**) Sequence alignment of the stalk of HN proteins from three different NDV strains and four other paramyxoviruses. Conserved residues (>70%) are boxed; completely conserved residues are highlighted in red. Fusion promotion residues are indicated by black asterisks. (**b**) Three-dimensional structure of the NDV-AV HN stalk generated by PyMOL software (Version 1.5, Schrödinger). Residues in the IR are displayed in ball and stick form, whereas the point mutations examined in this study are showed in sphere form. The structure was derived from the crystal structure of the NDV-AV HN protein reported by Yuan *et al.*^5^.

To compare the HN sequence of G7 with that of other related viruses, we performed amino acid alignment of the HN stalk domains of six selected paramyxoviruses. The HN stalk domain contains two HRs separated by an IR that harbors seven conserved amino acids (89-ALESPLA-95). P93 is absolutely conserved; however, the other residues are partially conserved among paramyxoviruses (Fig. 1a). Previous studies have demonstrated that mutations in the stalk (in or around the HRs and IR) can considerably impact the structure and function of HN^11^,^14^,^15^,^17^. The three-dimensional structure of the HN stalk generated by PyMOL software^5^ more clearly reveals the integral role of the HRs and IR in the formation of the 4 helical bundle (4-HB) (Fig. 1b). Recent research has demonstrated that substitutions of A89, P93, and L94 modulate the biological effectiveness of the BC strain^16^.

### Mutations in the IR of the G7 HN protein modulate NA activity, hemadsorption, and fusion promotion

**Figure 2:**
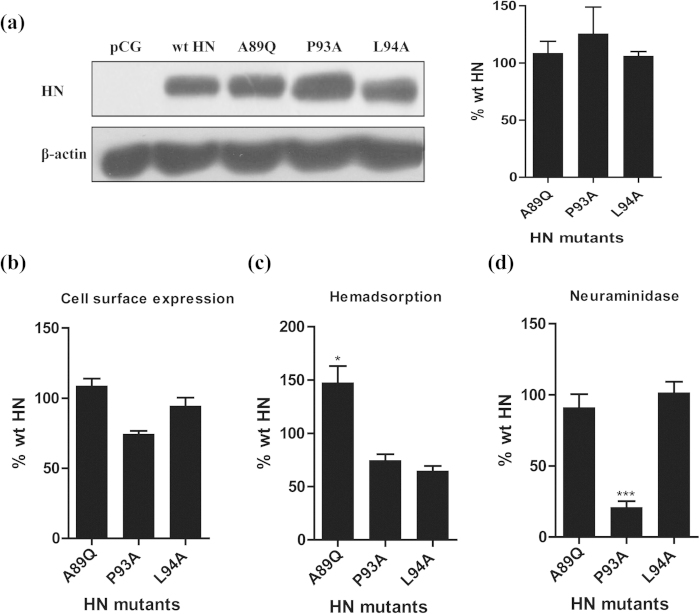
HN expression, HAd ability, and NA activity of wt HN and HN mutants. (**a**) pCG-HN **a**nd its mutants were constructed and transfected into BHK-21F cells. Total HN protein expression was verified by western blotting. (**b**) The amount of each HN protein at the cell surface (the cell surface expression; CSE) was determined by FACS analysis at 16 h post-transfection of BHK-21F cells by using anti-G7 serum. (**c**) The hemadsorption (HAd) activity was determined based on the ability of the HN expressed at the cell surface to adsorb chicken erythrocytes at 4 °C. (**d**) The neuraminidase (NA) activity was determined as the ability of the cell surface HA proteins to catalyze the release of sialic acid from 2’-(4-methylumbelliferyl)-α-D-N-acetylneuraminic acid. For all three of these assays, the background level obtained with vector alone was subtracted. All data are expressed relative to the amount of wt protein; bars represent the mean ± SD of three independent experiments (n = 3) (*P < 0.05, ***P < 0.001).

To further examine the importance of the A89, P93, and L94 residues, A89Q, P93A and L94A mutations were introduced into the HN gene of G7, and the effects were assessed at both the protein and virus levels. First, we tested whether these three mutations influenced the total protein expression of HN in BHK-21F cells. We found that the expression of the three mutants was similar to that of wild type (wt) HN (Fig. 1a). We then quantitated the cell surface expression of the mutant proteins by FACS analysis (Fig. 2b). The A89Q and L94A proteins were efficiently expressed relative to wt HN, suggesting that these two mutants are not misfolded. P93A was transported to the cell surface about 74.3% as efficiently as wt HN, which was not a significant difference in efficiency and was not prohibitive to additional functional testing.

To assess whether the three mutations affect G7 HN receptor recognition or NA activity, we transfected wt and mutant HNs into BHK-21F cells. P93A HN and L94A HN could hemadsorb at 64.5% and 74.4% of the wt level, respectively; however, A89Q HN had significantly increased hemadsorption (HAd) capacity (a 47.3% increase) compared with that of wt HN (Fig. 2c). In addition, the NA activity of A89Q was slightly weaker than that of wt HN, whereas the P93A mutation severely impaired the ability of HN to catalyze the release of sialic acid from the substrate, with only 21% of the wt level of NA activity (Fig. 2d).

**Figure 3:**
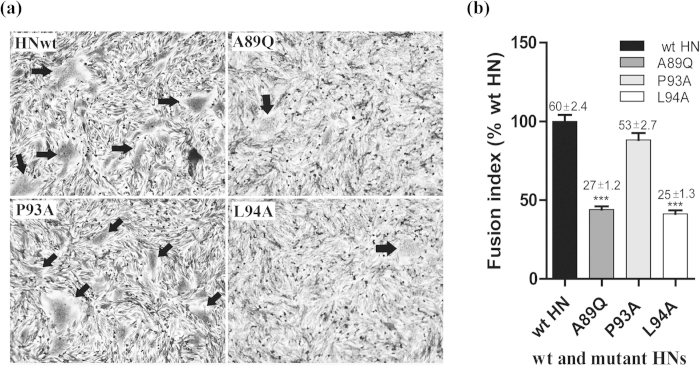
Syncytium formation in BHK-21F monolayers coexpressing HN and F proteins. (**a**) The extent of syncytium formation is shown for representative BHK-21F monolayers expressing pCAGGS (pCG) vector, wtHN, or HN bearing the A89Q, P93A, or L94A substitution. Cells were fixed with 4% paraformaldehyde and stained with Giemsa. Syncytia are indicated by black arrows. (**b**) The fusion index of wt HN and HN mutants was calculated as the mean number of nuclei per syncytium. All values are expressed relative to the HN wt value of 100%. The mean and standard error of 20 fusion events for each HN are shown above each column (***P < 0.001).

To further examine the effects of the three IR mutations, we evaluated their fusion promotion activities by measuring the ability of wt and mutant HN proteins to induce syncytium formation in conjunction with the homologous F protein in BHK-21F cells (Fig. 3). All three substitutions reduced the fusion promotion activity of the HN proteins in BHK-21F cells; however, the amount and size of the syncytium induced by the A89Q and L94A mutations were considerably lower than those induced by wt HN (Fig. 3a). Quantification of the fusion index (fusion promotion activity compared with the wt level) showed that the P93A mutation caused a slight decrease in fusion promotion activity (88%), whereas the A89Q and L94A mutants had sharply impaired fusion promotion activity (44% and 41%, respectively) (Fig. 3b).

These results suggest that each mutation has a specific effect on HN function, with the A89Q mutation causing increased receptor recognition capability but decreased fusion activity; the P93A mutation causing dramatic decreases in both NA activity and the receptor recognition capability; and the L94A mutation causing dramatic decreases in both receptor recognition and fusion activity.

### A recombinant virus bearing the P93A or L94A mutation, but not the A89Q mutation, is recoverable

To better understand the biological characteristics of these three mutations in the G7 HN gene at the whole virus level, we used plasmid-based reverse genetics to rescue individual viruses bearing the A89Q, P93A, and L94A mutations. In contrast to the results of a previous study that used a neurotropic BC strain^16^, recombinant viruses containing P93A and L94A were successfully recovered, but an A89Q virus was not recoverable. Thus, we were able to use two rescued mutant viruses (rG7-P93A and rG7-L94A) together with the rescued wild type virus (rG7-wt) for our subsequent experiments.

To examine genetic stability, the three recovered viruses were serially propagated for 10 passages in 9-day-old embryos, and their whole genomes were sequenced after the fifth and tenth passages. The results indicated that the P93A and L94A mutations in the HN gene were stable and that no other variations were present in the whole genome (data not shown).

**Figure 4:**
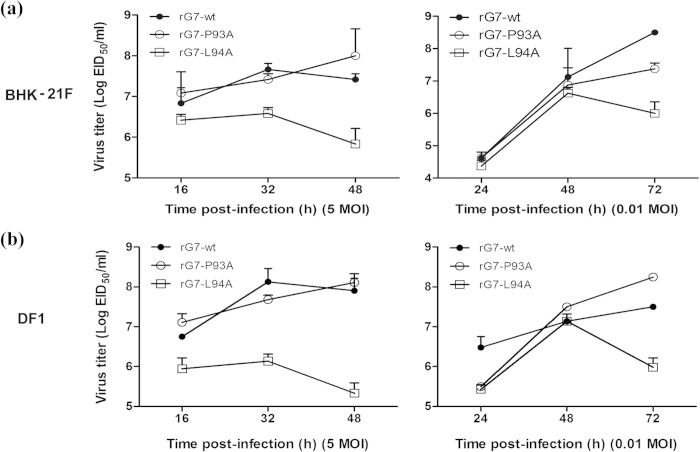
Growth kinetics of rescued wt and HN-mutated NDV viruses. The growth characteristics of viruses were determined by means of single and multi-cycle growth curves in both BHK-21F cells (**a**) and DF1 cells (**b**) infected with viruses **a**t MOIs of 5 and 0.01, respectively. Supernatants were collected at 8-h or 12-h intervals. Viral titers were quantitated in SPF embryonated chicken eggs. The data shown represent three independent experiments; bars represent the mean ± SD of three independent experiments (n = 3) (*P < 0.05, ***P < 0.001).

Next, we evaluated the growth kinetics of the recovered viruses by means of single and multi-cycle infections of BHK-21F and DF1 cells. BHK-21F cells were infected with rG7-wt, rG7-P93A, or rG7-L94A at a multiplicity of infection (MOI) of 5 or 0.01. When inoculated at an MOI of 5, rG7-wt reached a titer peak at 32 h post-infection (hpi), rG7-P93A grew more slowly but for longer than rG7-wt, and its titer was higher than that of rG7-wt at 48 hpi (Fig. 4). However, at an MOI of 0.01, the titers of rG7-wt and rG7-P93A continued to increase until the end of the observation period (72 hpi). The rG7-L94A virus was markedly attenuated, with a consistently low growth velocity in BHK-21F cells at 5 and 0.01 MOI. To confirm the growth of these viruses in avian cells, DF1 cells were infected with the three rescued viruses. The growth kinetics of three viruses was similar in BHK-21F and DF1 cells (Fig. 4b). These results indicate that the P93A mutation barely influences the growth of NDV in cells, but the L94A mutation severely disrupts growth.

### The P93A and L94A substitutions impair viral invasion, hemadsorption ability, NA activity, and fusion promotion in live viruses

**Figure 5:**
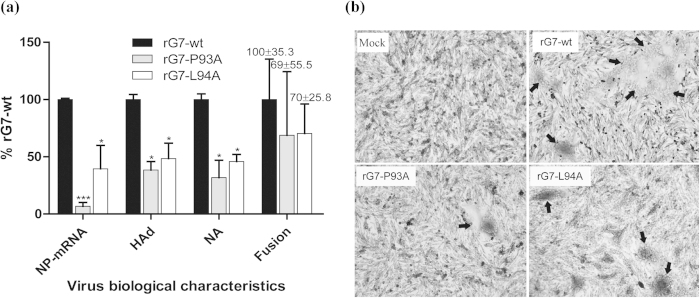
Biological characteristics of rescued wt and HN-mutated viruses. (**a**) The relative expression of the NDV NP gene, HAd ability, NA activity, and fusion index were assessed for virus-infected BHK-21F cells (MOI of 1). All values are expressed relative to that for rG7-wt, which was set at 100%. Each bar represents the mean ± SD of three independent experiments (n = 3) (*P < 0.05, ***P < 0.001). (**b**) Representative syncytium formation induced by viral infection of BHK-21F cells. Monolayers were fixed with 4% paraformaldehyde and stained with Giemsa. Syncytia are indicated by black arrows.

To analyze whether the P93A and L94A mutations modulate the biological activities of G7 in BHK-21F cells, the HAd, NA and fusion promotion activities of rG7-wt, rG7-P93A, and rG7-L94A were determined. Compared with rG7-wt, the mutant viruses had significantly reduced HAd ability and NA activity, which was consistent with the qRT-PCR results of virus replication in cells at the mRNA level (Fig. 5a). These results suggest that the P93 and L94 mutations severely impair viral entry and affect HN protein functions. The fusogenic abilities of the rG7-P93A and rG7-L94A viruses were approximately 70% of that of rG7-wt. Phenotypically, fewer syncytia were induced by rG7-P93A than by rG7-wt, whereas smaller syncytia were formed in the cells infected with rG7-L94A (Fig. 5b), which resulted in an earlier cytopathic effect and lower viral replication (Fig. 4).

### The P93A and L94A substitutions attenuate the replication and pathogenicity of G7 in specific pathogen-free chickens

To understand whether the different biological phenotypes of rG7-P93A and rG7-L94A *in vitro* correlate with pathotypes in embryonated chicken eggs or specific pathogen-free (SPF) chickens *in vivo*, we conducted three well recognized pathogenicity tests. As summarized in Table 1, the mean death time (MDT) of 9-day-old embryonated chicken eggs infected with rG7-P93A (56 h) or rG7-L94A (60 h) was prolonged compared with that for eggs infected with rG7-wt (44 h). The results of an intracerebral pathogenicity index (ICPI) test were consistent with the results of the MDT. Compared with the parental virus (ICPI = 1.7), rG7-P93A had a slightly decreased ICPI (1.56) and rG7-L94A had a markedly decreased ICPI (1.18). On the basis of both the MDT and ICPI values, the attenuated rG7-L94A virus would be classified as a moderately virulent strain. The results of an intravenous pathogenicity index (IVPI) test, which was evaluated in 6-week-old SPF chickens, showed that rG7-P93A and rG7-L94A had sharply decreased indices (0.76 and 0.58, respectively) compared with that of rG7-wt (2.4).

To provide further evidence that the P93A and L94A substitutions attenuate the virulence of G7 *in vivo*, we collected tracheal and cloacal swabs, and tissues from the spleen, intestine, and Bursa Fabricius from rG7-wt-, rG7-P93A- and rG7-L94A-infected groups of chickens on day 5 post-infection. As shown in Table 2, viral loads were observed in each of the swabs and tissues from the rG7-wt group and were much higher than those in the rG7-P93A and rG7-L94A groups. These results demonstrate that the P93 and L94 mutations impaired the viral infectivity and pathogenicity of the virus in chickens.

**Figure 6:**
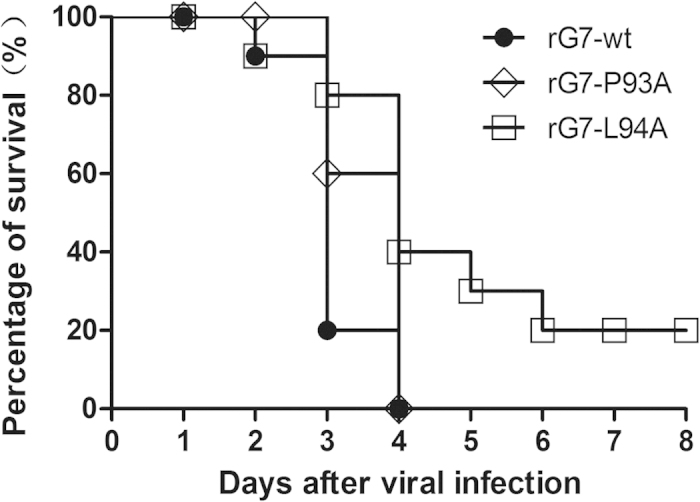
Survival of 1-day-old SPF chicks inoculated intracerebrally with rescued wt and HN-mutated viruses at 10^6^ EID_50_/chick. Ten chicks were allocated to each group.

To evaluate the overall effect of the P93A and L94A mutations on survival of infected chickens, survival curves for chicks infected with the three viruses were compared (Fig. 6). In agreement with the pathogenicity data, the rG7-P93A and rG7-L94A viruses displayed decreased virulence. On day 3 after inoculation, 80% of the rG7-L94A-infected chickens and 60% for the rG7-P93A infected chickens had survived, but the survival rate was only 20% for the rG7-wt-infected group. By day 4, there was 100% mortality for both the rG7-wt- and rG7-P93A-infected groups, but 40% of the rG7-L94A-infected chickens survived, and among those, 20% survived up to 8 days. These results are consistent with the *in vitro* growth kinetics data for the viruses and indicate that the P93A and L94A mutations impair infectivity and replication in chickens.

### Discussion

The NDV HN gene encodes a multifunctional protein that greatly contributes to virulence and pathogenicity^4^,^16^,^18^,^19^. Previous studies have demonstrated that mutations in the stalk region of the HN protein influence its biological characteristics to various degrees^11^,^14^,^15^,^20^. However, the results of these studies vary, possibly because of the use of different virus strains or technologies in different laboratories. In the present study, we chose three specific sites in the IR of the HN stalk to assess the function of the HN protein in the G7 epidemic strain and recovered viruses in cells and chickens for two of the mutations.

The three mutations we selected (A89Q, P93A and L94A) were chosen for their location within the IR and their conservation among viruses. Alignment of the HN sequences of the BC strain, a moderately virulent strain of genotype II, and G7, a more virulent strain of genotype VII, revealed that the protein identities are low (89%), especially for the F-specific HN stalk regions from amino acid 49 to 152, which has even lower identity (83%)^5^,^20^. Furthermore, previous studies suggest that even a single point variation in the HN or F protein can result in a large difference^15^,^21^. Therefore, this sequence diversity likely contributes to the variable outcomes for the protein and virus.

At the protein level, the three individual mutations in the IR had considerable influence on HN functions, confirming the belief that the IR is a vital region for NDV. The impairment of receptor recognition ability and NA activity induced by the P93A substitution was more dramatic in our hands than those of Iorio *et al.*, but the trend was similar^11^,^14^. Among the three mutations, P93A had the most significant impact on HN function, a finding that may be explained by the influence of proline on the formation of the α-helix secondary structure. However, the A89Q and L94A mutations did not significantly change the NA activity of the HN protein, but significantly impaired the fusion promotion activity, which might be attributed by the key role these two amino acid residues play in the interaction with the F protein^11^,^15^.

At the viral level, our data appear to contradict those of Kim *et al.*^16^. We were able to successfully rescue P93A- and L94A-containing viruses, but the A89Q mutant could not be rescued. These recovered viruses allowed us to re-examine the effects of mutation on the activity of the virus within a biological context. Notably, relative to wild type, both mutant viruses had reduced viral titers in chickens and attenuated effects on overall chicken survival.

NDV HN is a multifunctional protein with receptor recognition, neuraminidase activity, and fusion promotion activity. In the present study, we found several interesting correlations between HN functions and viral replication and pathogenicity in cells and chickens. P93A and L94-containing HN protein exhibited markedly decreased HAd ability and NA activity. Thus, the attenuated pathogenic phenotype of rG7-P93A and rG7-L94A in SPF chickens may be explained primarily by the effects of these mutations on receptor recognition and NA activity, even though the fusion promotion activities were only slightly impaired in terms of syncytium number and size at the virus level. Therefore, taken together, our data suggest that: i) mutations introduced into the NDV viral genome by using reverse genetics can be used to elucidate biological phenotypes; and ii) receptor recognition, NA activity, and fusion promotion are preferentially balanced at the protein and virus level and are the main determinants of the virulence and pathogenicity of rG7-P93A and rG7-L94A.

## Methods

### Sequencing and molecular modeling

Sequences editing, assembly, and analysis was performed by using Lasergene and ClustalX2. The three-dimensional structures of the HN protein (PDB code 3T1E) were downloaded from the PDB protein databank at http://www.rcsb.org/pdb/home/home.do and handled by the program PyMOL (DeLano, W.L. The PyMOL Molecular Graphics System (2002) DeLano Scientific, San Carlos, CA, USA.).

### Cells, viruses, and animals

BHK-21F cells were grown in DMEM supplemented with 10% FBS and penicillin-streptomycin (100 IU/ml). G7 is an epidemic strain in China belonging to class II, genotype VII. It was purified by using standard protocols and propagated in the allantoic cavities of 9-day-old SPF embryonated chicken eggs. The recombinant vaccinia virus expressing T7 RNA polymerase was kindly provided by Dr. Zhigao Bu (Harbin Veterinary Research Institute, CAAS). SPF eggs and chickens were purchased from Beijing Merial Vital Laboratory Animal Technology Co., Ltd. All infectious NDV experiments were conducted in a biosafety level-3 (BSL-3) laboratory. All animals were handled strictly according to the Good Animal Practice requirements of the Animal Ethics Procedures and Guidelines of the People’s Republic of China. This study was approved by the Animal Ethics Committee of Lanzhou Veterinary Research Institute, Chinese Academy of Agricultural Sciences (Approval No. SYXK2010-003).

### Plasmid construction and site-directed mutagenesis

The G7 virus stock was used for viral RNA extraction and reverse transcription into cDNA according to the instructions supplied with the commercial kits (Promega, Madison, WI, USA). The NP, P, F, HN and L gene segments were amplified by standard RT-PCR from the cDNA genome. The F and HN genes were inserted into a eukaryotic expression vector pCAGGs (pCG) between the Eco*RI* and Xho*I* restriction sites. The helper genes (NP, P, and L) were cloned into the pBlue-script KS(+) (pBSK) plasmid with Eco*RV*. Site-directed mutagenesis was employed to produce the HN mutations A89Q, P93A, and L94A with specific primers synthesized by Sangon Biotech Co., Ltd (Shanghai, China). The sequences of these recombinant plasmids were verified by sequencing (GENEWIZ Inc., Beijing, China).

### Cell surface expression

To quantitate the cell surface expression of G7-HN (wt HN) and its mutants, BHK-21F cells were seeded into six-well plates one day prior to experimentation. Each well was transfected with 2 μg of wt HN or HN mutants by using the Lipofectamine 2000 Transfectant Reagent (Invitrogen, CA, USA). At 16 h post-transfection, each well was incubated with G7 polyclonal serum at a dilution of 1:200 for 1 h at room temperature. The monolayers were then washed extensively with PBS to remove unbound antibodies and incubated with fluorescein isothiocyanate (FITC)-conjugated mouse anti-chicken IgG at a 1:500 dilution (Sigma-Aldrich, St. Louis, USA). Cells were washed extensively and resuspended in PBS containing 2% paraformaldehyde. The mean fluorescence intensity of 10,000 cells was recorded for each sample by using a FACSAria III flow cytometer (Becton Dickinson, New Jersey, USA)^11^.

### Virus recovery

G7 NP, P, and L genes were cloned into the pBSK vector as described above. To generate the full-length antigenome, five pairs of primers were designed and used to produce 5 subgenomic overlapping cDNA fragments, which were then ligated stepwise into a full-length genome cDNA and inserted into the pBSK plasmid under the control of a flanking T7 promoter and a stop sequence (pBSK-G7). By using specific primers, the required mutations were introduced into HN-containing fragments and placed within the genome to produce pBSK-G7/A89Q, pBSK-G7/P93A, and pBSK-G7/L94A. BHK-21 cells were seeded into six-well plates at 80%–90% confluence, and the vaccinia T7 recombinant virus was added at an MOI of 2. The plates were incubated at 37 °C for 1 h and then the cells were co-transfected with pBSK-NP, -P, -L, and full-length pBSK-G7 at a ratio of 1: 0.5: 0.5: 2. After 3 days of incubation, the cell supernatants were harvested, filtered, and then inoculated into 9-day-old SPF chicken embryos to rescue the viruses. After 3 days, the allantoic fluid was harvested and checked by using a hemagglutination (HA) assay. Viral RNA was extracted from the HA-positive allantoic fluid, and the entire genome cDNAs were generated and sequenced to confirm the presence of the desired mutations. The confirmed and purified viruses were stored at −80 °C as stocks for subsequent experiments^22^.

### Neuraminidase (NA) assay

The NA assay at the protein level was performed according to previously published methods^16^. The NA activities of the wild-type and rescued, recombinant NDV viruses were determined by using a modified fluorimetric assay. Briefly, viruses were inoculated at an MOI of 1 into confluent monolayers of BHK-21 cells grown in Opti-MEM at 37 °C. After 8 h, supernatants were collected from infected cells and serially diluted 2-fold in 32.5 mM MES (2-N-morpholinoethanesulfonic acid) buffer (pH 6.5) and mixed with 0.1 mM substrate (2’-[4-methylumbelliferyl)-α-D-N-acetylneuraminic acid) and 4 mM calcium chloride. After a 30-min incubation at 37 °C with shaking, the reaction was stopped by the addition of 0.014 M sodium hydroxide in 83% (vol/vol) ethanol. Fluorescence intensity was measured as described above (see Cell Surface Expression). Samples collected from virus-free cells served as controls. The NA values were normalized relative to 100% of the value for rescued G7-wt^23^.

### Hemadsorption (HAd) assay

The HAd assay at the protein level was performed according to published methods^12^. For rescued viruses, monolayers of BHK-21 cells were infected with the rescued viruses at an MOI of 1. After 8 h, the medium was decanted and the cells were washed with PBS. Then, hemadsorption was measured; the values were expressed relative to 100% of the value for rG7-wt^24^.

### NDV NP-specific RT-qPCR

BHK-21F cells were seeded in 12-well plates and were infected with each virus at an MOI of 1. After 8 hpi, cells were harvested at different time points for total RNA extraction with RNAiso Plus (TaKaRa, Dalian, China) and cDNA reverse translation (Promega, WI, USA). The intracellular mRNA level of the NDV NP gene was quantified by using a ROX Real-time PCR Kit (Roche, CA, USA)^25^. The specific primer sequences were designed by using Oligo7 software, and their amplification efficiency was tested before use in these experiments. The experiments were repeated three times. GAPDH served as an internal control, and the relative expression of rG7-wt was normalized to 100%.

### Fusion assay and syncytium formation of rescued viruses

BHK-21F cells in six-well plates were transfected as described above using 1 μg each of pCG-F and pCG-HN or the HN mutant plasmids. After distinct syncytium was observed, the cells were washed with PBS and fixed with 4% paraformaldehyde for 15 min. Then, the cells were stained with Giemsa and photographed under the microscope. Each virus was inoculated into BHK-21F cells at an MOI of 1. Fusion was quantitated by means of the fusion index, which is the ratio of the total number of nuclei to the number of syncytium in which the nuclei were counted. The values were expressed relative to the value for wt HN or rG7-wt, which was set at 100%.

### Growth kinetics of rescued viruses

The growth kinetics of rescued viruses (wt HN and HN-mutants) were determined under single and multiple-cycle growth conditions in both BHK-21F and DF1 cells. The viruses were inoculated at MOIs of 5 and 0.01 into cells grown in Opti-MEM at 37 °C. The supernatant was collected at different time points until 48 h or 72 h post-infection. The virus content in the samples was quantitated by determining the 50% egg infectious dose (EID_50_) in 9-day-old SPF embryonated chicken eggs. Briefly, samples were serially 10-fold diluted, and 100 μl of each serial dilution was injected into the allantoic cavities in triplicate. After 3 days of incubation at 37 °C, the allantoic fluid was harvested for the HA assay and the EID_50_ was calculated by using the Reed & Muench method^26^.

### Virulence of recovered viruses

The virulence of the recovered viruses was determined by: 1) the mean death time (MDT) in 9-day-old SPF embryonated chicken eggs; 2) the intracerebral pathogenicity index (ICPI) in ten 1-day-old SPF chicks; and 3) the intravenous pathogenicity index (IVPI) in ten 6-week-old SPF chickens. All tests were performed according to standard procedures^27^,^28^.

### Statistical analysis

Statistical differences between repeats were analyzed with GraphPad Prism 5 (GraphPad Software, California San Diego, USA) using ANOVA followed by Turkey’s test. Statistical significance was set at *P* < 0.05 (*) and *P* < 0.001(***), respectively.

## Additional Information

**How to cite this article**: Liu, B. *et al.* Two single amino acid substitutions in the intervening region of Newcastle disease virus HN protein attenuate viral replication and pathogenicity. *Sci. Rep.* **5**, 13038; doi: 10.1038/srep13038 (2015).

## Figures and Tables

**Table 1:** Pathogenicity of wild-type and HN-mutated viruses in SPF embryonated chicken eggs and chickens.

Virus	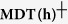	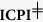	IVPI§
rG7- wt	44	1.7	2.4
rG7- P93A	56	1.56	0.76
rG7- L94A	60	1.18	0.58

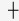
Mean death time (MDT) pathotype definition: virulent strains, <60 h; moderately virulent strains, 60 to 90 h; avirulent strains, >90 h.

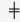
Intracerebral pathogenicity index (ICPI) is the criterion to classify the virulence of NDV isolates as recommended by the OIE. Pathotype definition: virulent strains, 1.5 to 2.0; moderately virulent strains, 0.7 to 1.5; avirulent strains, 0.0 to 0.7.

^§^Intravenous pathogenicity index (IVPI): velogenic strains yield values greater than 2.0, while avirulent strains approach 0.0.

**Table 2:** Tissue distribution of recovered viruses in 4-week-old chickens on day 5 post-infection.

Virus	Mean virus titer (log_10_ EID_50_/ml) ±SD				
	Trachea swab	Spleen	Intestine	Bursa Fabricius	Cloacal swab
rG7-wt	6.13 ± 0.53^a^	6.25 ± 0^a^	6.63 ± 0.18^a^	7.63 ± 0.18^a^	4.50 ± 0^a^
rG7-P93A	1.63 ± 2.30^b^	3.50 ± 0^a^	3.50 ± 0^b^	3.13 ± 0.53^b^	3.25 ± 0.71^a^
rG7-L94A	ND^b^	1.25 ± 1.77^b^	ND^b^	3.38 ± 1.24^b^	2.00 ± 0.35^b^

*4-week-old chickens were infected with the wt and mutated virus at 10^5^ EID_50_/chicken via the intranasal route. Two chickens per group were sacrificed on day 5 post-infection. Tissues were collected, and titers were determined by calculating the EID_50_ in SPF embryos. The titers are expressed as the mean ± SD (n = 3). ND, not detected. Different lowercase superscript letter indicates statistical differences (*P* < 0.05) within the column using Turkey’s multiple comparison test.
